# EASIER corpus: A lexical simplification resource for people with cognitive impairments

**DOI:** 10.1371/journal.pone.0283622

**Published:** 2023-04-12

**Authors:** Rodrigo Alarcon, Lourdes Moreno, Paloma Martínez

**Affiliations:** Computer Science and Engineering Department, Universidad Carlos III de Madrid, Madrid, Spain; STL UMR8163 CNRS, FRANCE

## Abstract

Thanks to technologies such as the Internet and devices now available to people, we have increasingly greater access to larger quantities of information. However, people with ageing disabilities or intellectual disabilities, non-native speakers, and others have difficulties reading and understanding information. For this reason, it is essential to provide text simplification mechanisms when accessing information. Natural Language Processing methods can be applied to simplify textual content and improve understanding. These methods often use machine learning algorithms and models which require resources, such as corpora, to be trained and tested. This article presents the EASIER corpus, a resource that can be used to build lexical simplification methods to process Spanish domain-independent texts. The EASIER corpus is composed of 260 annotated documents with 8,155 words labelled as complex and 5,130 words with at least one proposed context-aware synonym associated. Expert linguists in easy-to-read and plain language guidelines have annotated the corpus based on their experience adapting texts for people with intellectual disabilities. Sixteen annotation guidelines that discriminate between complex and simple words have been defined to help other groups of experts to generate new annotations. Additionally, an inter-annotator agreement test was performed to validate the corpus, obtaining a Fleiss Kappa coefficient of 0.641. Furthermore, a qualitative evaluation was conducted with 45 users (including people with intellectual disabilities, elderly people, and a control audience). Complex word identification tasks achieved moderate results, but the synonyms proposed to replace complex words achieved almost perfect ratings. This resource has been integrated into the EASIER platform, a tool that helps people with cognitive impairments and intellectual disabilities to read and understand texts more easily.

## Introduction

Information and communication technologies, especially the Internet, have transformed how we live and communicate. While millions of texts are produced every day, not all of these texts are easy to understand for everyone due to their complexity. Texts that contain unusual and complicated words can cause cognitive accessibility barriers for people with intellectual disabilities. In this sense, one solution can be to offer cognitively accessible interfaces and simplified text content, which benefit not only individuals with intellectual and learning disabilities but also deaf and deaf-blind individuals, the elderly, the illiterate and immigrants whose native language is different, among others. The need for simplified texts is becoming increasingly critical as the number of individuals with disabilities is growing due to the ageing population [1].

Manual production of simplified texts is a non-trivial and, at the same time, time consuming task [2]. In this sense, there are methods that systematically produce simplified content. Natural Language Processing (NLP) and artificial intelligence provide methods to simplify texts promoting readability and understandability for people with intellectual disabilities.

Some directives provide guidelines on making content more accessible for individuals with intellectual and learning disabilities. In this regard, the most important noteworthy initiatives are the Web Content Accessibility Guidelines (WCAG) [3], the Easy-to-Read guidelines [4–6], the Plain Language guidelines [7] and the document issued by the Cognitive and Learning Disabilities Accessibility Task Force (W3C-COGA TF) [8]. One specific guideline is frequently repeated in all these initiatives: use a simple lexicon. In addition, other guidelines indicate that providing synonyms for complex words is also beneficial. Therefore, providing simplified texts has been found to be helpful for people with intellectual disabilities from a lexical point of view.

Lexical simplification is an essential part of text simplification based on machine learning and deep learning methods to replace specific words with simpler ones for a particular audience. Lexical simplification requires a Complex Word Identification (CWI) task to detect words that are considered difficult for a target audience. Once these words are identified, Substitute Generation/Selection (SG/SS) tasks must offer a more straightforward synonym. SG tasks focus on producing substitutes for a target word in all the contexts in which it may appear. On the other hand, SS tasks collect these substitutes and select those that best fit the context in which the target word was found [9].

Although these methods have shown promising results, manually annotated data or corpora are required for training purposes. Unfortunately, for Spanish, few annotated texts are available. This lack of resources has become the motivation for this work.

In this article, the EASIER corpus is presented (https://github.com/LURMORENO/EASIER_CORPUS). This corpus aims to support CWI and SG/SS tasks, two important processes in text simplification aimed at an audience with intellectual disabilities. This has been achieved through the assistance of an expert linguist in easy-to-read and plain language guidelines. Two additional experts and people with intellectual disabilities have evaluated the resulting corpus to ensure the quality of the data provided.

The EASIER corpus has been integrated into the EASIER tool [10, 11] (https://github.com/LURMORENO/easier) (http://easier.hulat.uc3m.es/), that improves the readability and understandability of texts for users with intellectual disabilities.

This article is structured as follows. The “Background” Section introduces previous work related to corpora used in simplification tasks. The “Method” Section describes the steps and resources used to develop the corpus as well as the annotation guidelines. The “Corpus Description” Section provides some statistics of the corpus; the “User Evaluation” Section describes the experiments with different types of users. Finally, the “Conclusions” Section presents some conclusions and future work.

## Background

In 1996, the first automatic text simplification approach [12] performed a superficial analysis of texts to identify verbs and nouns in complex phrases. Syntactic simplification consists in identifying grammatical complexities in sentences and converting them into much simpler ones [13]. The case of lexical simplification, which is the focus of this work, consists of substituting words in a given phrase to make it simpler without modifying its syntactic structure in any way.

The PSET project [14] aimed to create a system that performs lexical and syntactic procedures to assist people with aphasia in reading English newspaper texts. In Portuguese, the PorSimples project [15] developed technologies aimed at improving web content for people with low literacy levels by performing lexical/syntactic modifications and, at the same time, developing resources for this language, such as a parallel corpus with simplified sentences. For the French language, works based on parallel corpora such as the Alector corpus [16] have been presented, which focus on alleviating reading difficulties for people with low reading level or people with dyslexia. Additionally, French domain-specific resources have been proposed, such as the CLEAR corpus [17], which contains parallel instances of medical terms with their simplified version, aiming to alleviate the difficulty present in text with specialized content. The Simplext project [18, 19] worked on Spanish texts using a modular system for lexical and syntactic procedures to help people with cognitive disabilities. The FIRST project [20] was focused on developing language technologies to help autistic people, relying on a set of rules, images and dictionary searches for document simplification. Moreover, for people with intellectual disabilities, an accessible web e-mail client that performed text simplification was developed in the Able2Include project [21] to address web text accessibility in the context of e-mail communication. More recently, the authors in the EASIER project developed a web application that provides people with an easier way to improve the readability and comprehension of texts in Spanish. This work has been carried out with the objective of providing relevant data to improve lexical simplification [10, 22, 23].

Text simplification has been approached from different perspectives: using rule-based or machine learning systems to identify and improve complex texts [24]. Currently, deep learning systems are being used to generate a simplified version of a given text in a kind of machine translation process, see [25] for a comprehensive state of the art in text simplification. No matter what type of system is being used, it is always necessary to have resources to build, train or adapt text simplification methods. Annotated and simplified corpora are an essential part of these resources in NLP systems development.

Parallel corpora, which contain original texts together with their simplified versions, are very valuable resources for training text simplification algorithms, especially in languages with few resources, as is the case of Spanish. There are parallel corpora with aligned texts with a range of complexity levels; Table 1 shows some examples of relevant related resources in text simplification for English and Spanish.

**Table 1:** Text simplification resources for English/Spanish.

Resource	Annotated text	Size	Language: English (EN) Spanish (ES)	Annotation method
Simple English Wikipedia	A simplified version of regular Wikipedia	183,000 content pages to date	EN	Pages edited by 1,203 active users
SemEval 2012 [34]	English Internet Corpus [35]	2,010 instances of simplicity rankings	EN	Native English speakers
LSeval [36]	English Internet Corpus [35]	430 instances of simplicity rankings	EN	46 Amazon Mechanical Turk (turkers), 9 PHD students
LexMTurk [37]	Wikipedia	500 instances with target complex words and simpler synonyms	EN	50 Turkish English speaking
BenchLS [38]	Compilation of LSeval and LexMTurk	929 instances with an average of 7 candidates per complex word	EN	Corrected and filtered by English speakers
NNSeval [39]	Filtered version of BenchLS	239 instances	EN	Non-native english speakers
Wikipedia—Simple Wikipedia	Simple English Wikipedia	167,689 aligned sentences	EN	Language modelling [40]
PWKP (WikiSmall) [41]	Wikipedia and Simple Wikipedia	108,016 aligned sentences	EN	Statistical machine translation
Simplext [19]	News texts	200 aligned news texts	ES	Human editors trained in easy-to-read guidelines
SS Corpus [42]	Wikipedia and Simple Wikipedia	492,993 aligned sentences	EN	Unsupervised method
Newsela [28]	News articles	Parallel simple-complex articles with 11-grade levels	EN, ES	Manually produced by professional editors
RANLP 2017 [43]	Wikipedia	14,280 instances with target complex words	EN, ES	54 turkers (Native and non-native speakers)
WikiLarge [44]	WikiSmall, Aligned sentences pairs [40, 45]	2,000 for dev, 359 for test, 296,402 for training	EN	Combination of previously created simplification corpora
PPDB-S/M [46]	PPDB	5,709 unigrams for S size, 15,524 unigrams for M size	ES	Built by filtering and ordering paraphrases pairs from the paraphrases database (PPDB)
CASSA [46]	CASSA dataset	5,640,694 5-grams	ES	Generated by extracting all unique 5-grams pairs from CASSA resource
ASSET [47]	TurkCorpus extension	23,590 human simplifications associated with 2,359 sentences from TurkCorpus	EN	Amazon Mechanical Turk
VYTEDU-CW) [48]	Transcripts of academic videos	9,175 words, 723 annotated as complex	ES	430 annotators students
ALEXSIS [49]	RANLP 2017 datasets	381 instances with an average of 10.28 substitutes per instance	ES	prolific.co annotators

The most common are corpora comprised of a set of original sentences and their simplified versions. The Simplext project provided new resources such, as a parallel corpus comprised of 200 news texts, including their original and simplified versions. Other examples are [26–28] in English, [29] in Portuguese, [30] in German, [31] in Italian and [18, 28, 32] in Spanish. A recent paper [33] presents an overview of parallel corpora for text simplification in different languages, which complements the contents of Table 1.

Regarding lexical simplification, specific resources have been made available over the years. In English, SemEval-2012 [34] provided possible substitutes for a target word ranked in ascending order by their complexity, taking the context into consideration or based on the lexical substitution dataset [50], which focused on finding the best set of candidates for the substitution of a target word. Other resources were created using alignment methods. Horn et al. [37] created a collection of 500 sentences, which became a crowd‐sourced lexical substitution resource sampled from English Wikipedia and Simple English Wikipedia alignments. In Spanish, Baeza-Yates et al. [24] automatically created a database from the Spanish Open Thesaurus and the 5-gram Google Books Ngram Corpus. This resource was then extended in the work of Štajner et al. [46] by combining it with other resources such as OpenThesaurus (https://web.archive.org/) and EuroWordnet (https://archive.illc.uva.nl/EuroWordNet/). Also, certain resources were given additional specific tasks. For English CWI, in SemEval-2016 [51] a set of instances were presented, each of which had metadata associated with a target word labelled as either simple or complex. Some years later, the same task for English, Spanish, German and French was proposed [43], with the added value of performing classification for uni-words and multi-words. Recently, the ALEXSIS dataset [49] exploited the data from this task to create a new dataset containing simplicity-ranked substitutes for complex words. Also, a recent workshop [48] proposed a resource by challenging the participants to perform the CWI in academic content. Therefore, the proposed systems had to detect which technical words are commonly used in the domain and labelled them as simple words.

Most of these resources have been labelled by annotators without knowledge about cognitive accessibility, easy-to-read and plain language guidelines. Also, people with disabilities are not taken into account in the annotation process as is indicated in the “Annotation method” column in Table 1. EASIER corpus addresses this gap providing support for the CWI task and searching the corresponding synonym aimed at people with cognitive impairments, such as the elderly and people with intellectual disabilities, among others. The EASIER corpus has been annotated by easy-to-read and plain language experts following a methodological approach that involves people with disabilities.

## Method

Before explaining the methodology, recruitment of annotators, materials and instruments, it is important to mention that the experiments presented in this article have been reviewed to ensure that no confidential information is disclosed and has been approved in written form by an IRB at Universidad Carlos III de Madrid (IRB20_12) on October 28, 2020 and by the participants at subsequent dates.

### Selection of annotators

Three annotators have taken part in corpus construction. One annotated the entire corpus (main annotator), while the other two annotated part of the corpus to calculate the Inter-Annotator Agreement (IAA). The three annotators are Spanish native speakers, expert linguists and specialists in easy-to-read and plain language guidelines. They have more than 15 years of experience transforming conventional texts into easy-to-read texts. They belong to Plena Inclusión (https://plenainclusionmadrid.org/) Madrid and Grupo Amas Fácil (https://amasfacil.org/), two organisations that work to offer resources adapted to people with intellectual and learning disabilities. It should be noted that these annotators manually adapted the texts following a methodology that involves people with intellectual disabilities throughout the process.

### Materials

Two hundred and sixty news articles from the “60 y más” magazine (http://www.revista60ymas.es/60mas_01/index.htm), ranging from beginning of 2019 until the first months of 2020, were randomly selected based on their length. News covered a range of different topics in the areas of current affairs, health, guides for seniors and news. Thus, the EASIER corpus is a domain-independent corpus. Each document had a similar length, and the corpus has an average of 15 sentences per document. This journal belongs to Imserso (https://www.imserso.es/imserso_01/index.htm), the Institute for the Elderly and Social Services in Spain. This group’s main objective is to promote the social integration of the elderly through information in Spanish.

### Instruments

Annotators used an annotation tool created as an extension for Google Chrome (https://github.com/ralarcong/EASIER_AnnotationTool). The authors have developed it to (a) select and deselect words that are considered complex or unusual in a given text and (b) propose simple, context-appropriate synonyms for the target word.

**Fig 1:**
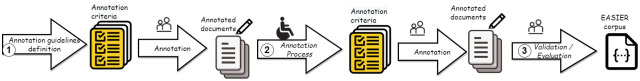
Corpus building methodology.

The corpus construction methodology includes three steps following an iterative process (see Fig 1):

1. **Annotation Guidelines Definition**. Based on the annotator’s experience and knowledge of easy-to-read and plain language guidelines, the main annotator establishes various annotation guidelines to detect complex words and suggest simple synonyms.
2. **Annotation Process**. The annotator performs the analysis of the texts according to the annotation guidelines using the annotation tool.
3. **Annotation Guidelines Validation**. In order to validate the annotation guidelines, an initial evaluation with the participation of people with intellectual disabilities of the set of texts annotated to date was performed. Once the documents have been fully annotated, the resulting corpus is described in the “Corpus description” Section. A portion of the data set is extracted and annotated by two other annotators to calculate IAA.

The annotation process, which describes the steps of the methodology, is shown below.

### Annotation guidelines definition

The main annotator defined the annotations guidelines and annotated complex words in texts accordingly. The terms given below should be annotated as complex terms:

1. Words that are common in verbal communication but probably are unknown to the people under study. The Spanish linguistic frequency indexes (Gran Diccionario de Uso del Español Actual, Corpus CREA (https://corpus.rae.es/lfrecuencias.html), Corpus CORPES XXI (https://www.rae.es/banco-de-datos/corpes-xxi) [4, 6, 52–56] are the resources used to identify these words.
2. The syllable configuration of a word should also be considered. When syllables are long or have more consonants, the effort needed to pronounce them could affect comprehension [6, 54, 56, 57].
3. Long words that are difficult to read and pronounce such as “esternocleidomastoideo”, (sternocleidomastoid), represent difficulty in reading and pronunciation [6, 56].
4. Technical jargon, for example, terms used in the medical or legal fields [4, 6, 55, 56].
5. Abbreviations or acronyms when an explanation is not included in the document. For example, a document explaining the objectives of the WHO, but the expansion “World Health Organization” is not included in the text [4, 6, 55, 56, 58].
6. Words in a language other than the main language of the document. Since EASIER’s target audience is the elderly and people with disabilities, it should not be assumed that they know other languages [4, 6, 56].
7. Roman numerals [6, 56, 59].
8. Idioms because they could have a double meaning that is difficult to understand, such as “cost an arm and a leg” which gives the sense of something expensive [6, 56].
9. Metaphorical expressions because are hard to understand [4, 6, 56].
10. Abstract terms which physical form cannot be perceived or imagined. For example, Terms such as “justice” or “emotion” are considered difficult to understand [4, 6, 56].
11. Multi-word terms of different types [4, 6, 56]:
  - Expressions constructed with complex words. For example, “key indicators” or “contractual resources”.
  - Expressions including simple words whose more familiar meaning has been modified. For example, “social tourism” or “portfolio of services”.
  - Complex expressions including complex and simple words whose most well-known meaning has been modified. For example, “strategic framework” or “inter-territorial council”.
12. Common words whose most frequent meaning is modified by the context in which they are found (linked to polysemy). For example, the “active” word has two senses: (a) the portion of the population either with a job or looking for a job and (b) a person who likes to be active, being the most used the second one [6, 56].
13. Percentages and mathematical expressions, for example, numbers expressing largequantities [4, 6, 56, 60].
14. Adverbs ending in “-mente” (-ly) because of their prolonged pronunciation [6, 56].
15. Collective nouns because are harder to understand than enumeration. For example, the concept “indumentaria” (clothing).
16. Words that are obsolete or in disuse [56].

The Table in S1 Table shows examples of selected uni-words or multi-words according to the criteria described in this section are provided.

### Annotation guidelines validation

A quarter of the dataset was annotated to assess the initial set of annotation guidelines, and a set of experiments were carried out with people with cognitive disabilities belonging to the target group. The aim was to evaluate and refine the expert linguist’s annotation guidelines.

The participants, the methodology and the results of this validation are explained below.

#### Participants

Some validation sessions were held in which people with disabilities are the validators to ensure that the adaptation is being done correctly. Eight people with mild intellectual disabilities (Group 1) and older people (Group 2), with five women and three men were chosen to participate in the initial evaluation. Of the five women, three were people with intellectual disabilities and two were elderly. In the group of men, two were people with intellectual disabilities, and one was an older adult. The validators’ age ranged from 25 to 86, seven with primary education and one with secondary schooling.

#### Methodology

The method used to validate easy reading texts by people with intellectual disabilities is supported by results reports from European projects such as the train2validate project (https://plenainclusionmadrid.org/train2validate/?lang=es), Pathways project (https://www.inclusion-europe.eu/pathways-2/), and complies with standards such as *Guidance on making written text easy to read and easy to understand* [61] and *Easy to read. Guidelines and recommendations for elaborating documents* [6]. This validation is organized in group sessions with a facilitator, support professional, and people with intellectual disabilities who participated as validator because they have reading comprehension difficulties. The validation session lasted three hours, including a twenty-minute break, and was moderated by a facilitator and our expert in easy-to-read who was annotating our corpus. The validators were provided with documents containing twenty-five complex words. These documents belong to the current affairs section (see Table 2), all framed within sentences and the corresponding synonyms. The moderator projected the document on a screen, then read each sentence aloud and asked the group whether they knew the adverse word or not and its meaning. This was an important step that allowed for assessing the participants’ comprehension capacity and clarifying the concepts if there were doubts. Each validator gave his or her opinion and was free to make comments as they saw fit. The moderator then read the synonyms and reread each sentence aloud, substituting each synonym’s adverse word. Finally, the validators commented on the meaning of each synonym, determined the most appropriate option and, if there were several synonyms, ordered them according to their comprehension criteria, which are as follows:

- Known for both groups: Every validator understands the meaning of the word.
- Explanation required: Every validator has an idea of the meaning of the word due to its context but at least one of them needs an explanation.
- Unknown: At least one validator does not know/understand the word.

**Table 2:** An extract of the target/synonym dataset for human evaluation with Group 1 (people with mild intellectual disabilities) and Group 2 (older people).

Target Word	Synonyms	Conclusion
Etiquetado (Labelling)	Letrero (sign), inscripción (inscription), rótulo (banner)	Explanation required for both groups
Etiqueta (formal/label)	Ceremonia (ceremony), protocolo (protocol)	Explanation required for Group 1 Known Group 2
Envasados (packaged)	Empaquetados (packaging)	Known by both groups
A granel (in bulk)	Suelto (loose), sin envase (without packaging)	Known for both groups
On-line (Online)	en línea (online), conectado a Internet (connected to the Internet)	Known by Group 1 Explanation required for Group 2
Comensales (diners)	Invitados (guests)	Unknown by Group 1 Known by Group 1
Saludables (salubrious)	Sanos (healthy), beneficiosos (beneficial)	Explanation required for both groups
Copiosa (copious)	Abundante (abundant)	Unknown by both groups
Crudos (raw)	sin cocinar (not cooked)	Known by both groups
Denominación (denomination)	Nombre (name)	Explanation required for both groups
Reclamar (claim)	Demandar (sue), quejarse (complain), exigir (demand)	Explanation required for both groups
Irregularidades (irregularities)	Anomalía (anomaly), alteración (alteration), variación (variation)	Unknown by both groups
Óptimas (optimum)	Buenas (good), excelentes (excellent)	Explanation required by both groups
Embalaje (packaging)	Envase (container), envoltorio (wrapping)	Known for both groups
Íntegro (exhaustive)	Entero (whole), completo (complete), intacto (intact)	Known for both groups
Consumidor (consumer)	Comprador (buyer/purchaser), cliente (client), usuario (user)	Explanation required for Group 1 Known Group 2
Provisional (provisional)	Temporales (temporary)	Unknown for both groups
Consejo (Council)	Asambleas (assembly), juntas (board), comisiones (commission/committee)	Known for both groups
Proporcionar (provide)	Dar (give), proporcionar (provide)	Known for both groups
Ciudadanía (citizens)	Sociedad (society), población (populace), nacionalidad (nationality)	Known for both groups
Veraz (veracious)	Real (real), cierta (certain), verdadera (true)	Unknown by both groups
Eficacia (efficiency)	Utilidad (usefulness), efectividad (effectiveness)	Unknown by both groups
Contrastar (contrast)	Comprobada (proven), comparada (compared), verificada (verified)	Unknown for both groups
Soporte (base)	Base (basis), fundamento (foundation), apoyo (support)	Unknown for both groups
Evidencias (evidence)	Certeza (certainty), seguridad (security), prueba (proof), demostración (demonstration)	Known for both groups

#### Results and discussion

Table 2 shows a portion of the dataset used for evaluation. The human evaluation showed that most of the words represented a challenge for the participants to comprehend (84%), either because they were unfamiliar with said words or needed additional explanation by the moderators. This demonstrates moderate results regarding the quality of the corpus in the decision making of word complexity criteria. For the synonyms proposal, the validators responded well, showing a better understanding of the text with the proposed synonyms. However, users gave a different priority to the suggested synonyms. For example, they understood the word “alteraciones” (alterations) better than the word “irregularidades” (irregularities). Also, users experienced increased difficulty understanding when more than three synonyms were proposed. Thanks to the validation session, the need for several resources or elements to assist in understanding the meaning of a complex word was confirmed. In some cases, it was found that merely showing possible substitutions for a word was not enough for participants to fully understand it, as the user required additional information about the word, such as a definition or an example. This requirement reaffirms the objectives of the EASIER project within which this work is framed. In addition to satisfying the processes of lexical simplification (CWI, SG/SS), this project offers additional comprehension aids such as providing disambiguated definitions and pictograms [10, 62].

## Corpus description

A total of 260 documents were annotated with complex words, from which an average of 15 sentences per document was obtained. As a result, approximately 8,100 complex words were gathered. At the same time, it should be mentioned that more than 5,100 words, for which at least one synonym was proposed, were also obtained (see Table 3).

**Table 3:** EASIER corpus statistics.

	EASIER
Documents	260
Sentences	3,778
Words	134,528
Average number of sentences per document	15
Average number of tokens per document	517
Total instances for CWI	44,975
Complex Words	8,155
Total instances for SG/SS	5,130
Proposed synonyms	7,892
Average of complex Words per document	30
Average of proposed synonyms per document	29
Complex Words with at least one substitute	5,130

Two distinct datasets could be distinguished: one for Complex Word Identification (CWI) tasks and another for Substitute Generation/Selection (SG/SS) tasks. Each instance of the CWI dataset has six columns (See Table in S2 Table) and are represented as follows:

1. The first column shows the ID of the document.
2. The second column shows the ID of the sentence for a particular word.
3. The third column shows the sentence.
4. The fourth and fifth columns show the offset of the target word.
5. The sixth column shows the target word.
6. The seventh column shows the correct label for the binary task (0: simple or 1: complex).

For the second dataset, each instance has five columns (See Table in S3 Table) and are represented as follows:

1. The first column shows the ID of the document.
2. The second column shows the ID of the target word.
3. The third column shows the target word.
4. The fourth column shows the sentence.
5. The fifth column shows the suggested synonyms for the target word separated commas.

### EASIER corpus dataset evaluation

In order to determine how well an annotation task is defined, the IAA is used to show how individual annotators compare to each other. This has been done for the CWI adm SG/SS datasets as is explained below.

#### Complex Word Identification (CWI) dataset inter-annotator agreement

Two additional annotators performed the agreement. First, for the CWI dataset evaluation, the decision was made to evaluate the Fleiss Kappa coefficient since it is intended for assessments carried out between two or more annotators. However, to obtain a more in-depth analysis between scorers, the Cohen’s Kappa coefficient between each annotator has been evaluated.

Following corpus annotation recommendations [63], to evaluate complex words’ annotation, 10% of the corpus was randomly extracted. As a result, 26 documents were obtained, from which 390 sentences to evaluate were obtained. As can be seen in Table 4, these metrics were extracted based on the POS tags, e.g., in the case “N” only metrics were calculated for the nouns of the corpus instances, while for “N—V—A”, they were calculated for the noun, verb and adverb tags as a whole (full evaluation can be found at https://github.com/ralarcong/EASIERCORPUS_EVALUATIONS).

**Table 4:** EASIER corpus—CWI dataset results where N: nouns, V: verbs, A: adverbs, I: Interjections, PN: proper nouns, M: multi- words.

POSTAG	Cohen’s Kappa (Rater 1–2)	Cohen’s Kappa (Rater 1–3)	Cohen’s Kappa (Rater 2–3)	Fleiss Kappa
**N**	0.4750	0.4114	0.5711	0.484
**V**	0.4082	0.5218	0.4385	0.454
**A**	0.2011	0.1942	0.4640	0.31
**I**	0.5002	0.1545	0.2658	0.3
**PN**	0.2263	0.2441	0.5338	0.347
**N—V**	0.4667	0.4365	0.5586	0.487
**N—V—A**	0.4628	0.4374	0.5602	0.487
**N—V—I**	0.4689	0.4342	0.5559	0.486
**N—V—I—PN**	0.4330	0.4228	0.5530	0.471
**N—V—M**	0.6455	0.6079	0.6728	0.641
**N—V—A—M**	0.6422	0.6094	0.6739	0.641
**N—V—I—M**	0.6465	0.6060	0.6707	0.64
**N—V—I—PN—M**	0.6067	0.5926	0.6597	0.619

According to the analysis of results, a moderate result was obtained with a Fleiss Kappa coefficient of 0.641. The highest agreement was reached when analysing the multi-words since long words or phrases make it difficult to understand the message. On the other hand, interjections were considered to have lexical content in some cases. Therefore, these few instances are removed from the corpus.

#### Substitute Generation/Selection (SG/SS) dataset evaluation

Inspired by previous work [64–66], a scale-based methodology was used to evaluate the content of the synonym dataset. The original annotator proposed synonyms for a target word and did not assign labels for this dataset. Therefore, to evaluate this dataset and in order to verify the quality of the proposed synonyms, the two additional annotators were asked to assign two types of labels for each synonym: “0: synonym incorrectly defined” and “1: well-defined synonym”. To this end, 10% of the total number of instances were extracted in which the target word needed to have at least three proposed synonyms. As a result, a dataset of 513 target words was obtained together with their respective synonyms.

**Fig 2:**
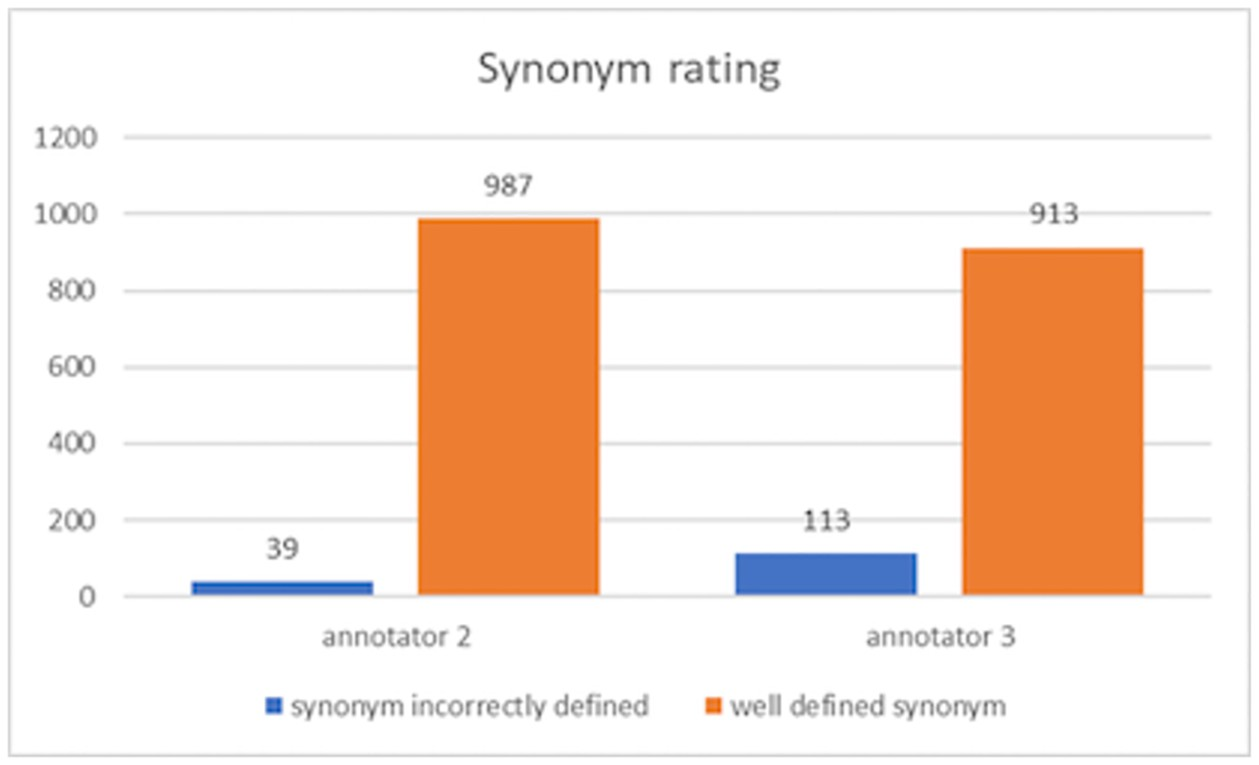
Annotations between annotator 2 and 3.

Fig 2 shows that positive results were obtained, as evidenced by the clear difference between well-defined and incorrectly defined synonyms. Of the 1,026 synonyms reviewed, annotator 2 rated 987 synonyms as well-defined and 37 as incorrectly defined. In turn, annotator 3 rated 913 synonyms as well-defined and 113 as incorrectly defined. Subsequently, an analysis was carried out of the instances in which the synonyms were rated as incorrectly defined. It was found that in several cases, these words were qualified in this way due to the fact that, although they could fit in the context, they presented some ambiguity with regard to their meaning. An example of this is the word “salubrity” in the sentence “Tiempos en los que la salubridad era escasa.” (Times when salubrity was scarce). The well-defined replacements were “limpieza” (cleanliness) and “hygiene” (hygiene). However, the incorrectly defined replacement was “salud” (health), which may work within the context of the sentence but modifies its semantics.

## User evaluation

In this section, different experiments to validate the EASIER corpus are described including participants, materials, procedure, tasks and metrics used for each experimentation (also available at https://github.com/ralarcong/EASIERCORPUS_EVALUATION).

### Participants

A total of 45 participants were recruited for this experimental study. The inclusion criteria were people with cognitive disabilities that included people with mild cognitive impairments medically identified and older people who have cognitive problems due to age deterioration. In addition, people without disabilities as a control group were considered. The participants were recruited by the HULAT group (https://hulat.inf.uc3m.es/) to which the authors belong in collaboration with the AMAS group (https://www.fundacion-amas.org/), an organization that works to provide resources for people with intellectual disabilities.

Table 5 shows an overview of the demographic information of the participants. The participants were divided into three groups: Group 1 represented 15 older people (33.3%), Group 2 represented 15 people with intellectual disabilities (33.3%) and Group 3 represented 15 control users (33.3%).

**Table 5:** Participant demographic information for corpus study (Group 1: Elder people, Group2: People with intellectual disabilities, Group 3: Control users).

Features	Group 1		Group 2		Group 3		All participants	
	N = 15	%	N = 15	%	N = 15	%	N = 45	%
**Age**								
71+	7	(47)	-	-	-	-	7	(16)
45–70	8	(53)	4	(27)	3	(20)	15	(33)
34–44	-	-	5	(33)	5	(33)	10	(22)
33 or younger	-	-	6	(40)	7	(47)	13	(29)
**Gender**								
Male	7	(47)	6	(40)	8	(53)	21	(47)
Female	8	(53)	9	(60)	7	(47)	24	(53)
**Education (Highest completed)**								
None	1	(7)	2	(13)	-	-	3	(7)
Primary school	5	(33)	7	(47)	-	-	12	(27)
High school	9	(60)	6	(40)	5	(33)	20	(44)
University education	-	-	-	-	10	(67)	10	(22)
**Reading experience (books per year)**								
None	3	(20)	9	(60)	3	(20)	15	(33)
1–3	5	(33)	3	(20)	6	(40)	14	(31)
3–6	4	(27)	1	(7)	4	(27)	9	(20)
6–12	3	(20)	2	(13)	1	(7)	6	(13)
12+	-	-	-	-	1	(7)	1	(2)

Across the entire population (all groups), the lowest number of participants corresponded to the age group between 34 and 44 years old with 10 participants (22%) and to participants over 71 years old with 7 participants (16%); on the other hand, the highest number of participants corresponded to the age group under 33 years old with 13 participants (29%) and to participants between 45 and 70 years old with 15 participants (33%).

There was a small difference between the number of female (53%) and male (47%) people with 24 and 21 participants respectively.

Regarding the educational level of the participants, the least number of participants were registered for people with no registered studies and people with a university degree with 3 (7%) and 10 (22%) participants respectively, and the majority had a high school level of education with 20 participants (44%), followed by primary level with 12 participants (27%).

Finally, the reading level of the participants was evaluated through the number of books read per year, where the lowest number of participants was concentrated by 1 (2%) participant who read more than 12 books per year, 6 (13%) participants who read 6 to 12 books per year, followed by 9 (20%) participants who read 3 to 6 books per year. While the highest number of participants was presented by participants who do not read any book per year and participants who read 1 to 3 books per year with 15 (32%) and 14 (31%) participants respectively.

### Materials

For this experimental study 29 sentences of similar length were randomly extracted to evaluate the detected complex words and suggested replacements.

### Procedure

The ethical committee of the Universidad Carlos III de Madrid (IRB20_12) approved this experimental study for people with and without disabilities on October 28, 2020. Participants were briefed on the purpose of the experiment and signed a consent form. In the case of people with intellectual disabilities, permission was obtained from their legal guardians. Next, participants were asked to complete a simple demographic questionnaire. Finally, each participant was asked to complete the tasks.

The validation method used with people with intellectual disabilities was similar to the initial evaluation of the corpus, described in the Annotation Guidelines Validation section. The sessions were conducted at the AMAS Group facilities, where the researcher worked together with the AMAS facilitators. The rest of the tests were carried out at the university facilities, where the researcher worked directly with the user.

The main steps were:

1. Demographic questions about age, gender, education level and reading habits.
2. Explanation and performance of task 1, referring to the CWI task.
3. Explanation and performance of task 2, referring to remaining tasks in the lexical simplification process where a substitute is provided by the EASIER corpus.

### Tasks

To evaluate the corpus, the following tasks were defined.

1. **Task 1** aims to measure the CWI task, i.e., the annotations of the corpus when discerning between complex and simple words. Each participant had to analyze 14 randomly selected sentences. In each sentence, the participant had to select single or multi-words that he/she judged to be complex or difficult to understand.
2. **Task 2** aims to measure the quality of the synonyms of the detected complex words, in order to determine whether the synonyms proposed by the EASIER corpus actually help to improve the cognitive comprehension of the texts. Each participant had to analyze 15 sentences, randomly selected. In each sentence, a detected complex word is highlighted and three candidate synonyms retrieved from the corpus are suggested. Thus, each participant had to analyze the sentences with each candidate and, as a next step, answer yes/no questions about whether the candidate helped to further understand the sentence.

### Measures

The measures in this experimentation were metrics used in the field of machine learning methods in order to compare the proposal with other related works [9, 38], which are the following:

- **Accuracy**: Represents the amount of correct identified words among all words.
- **Precision**: Amount of positives that are true.
- **Recall**: Amount of complex words correctly captured.
- **F-1**: The harmonic mean between precision and recall

In addition, different statistical metrics were used to obtain statistical significance, which are described in the next section.

### Results and discussion

This section gives results and discussions of the experiments described above. Likewise, this section is divided by the type of experimentation, complemented by subsequent analysis.

Table 6 shows the scores for task 1. The results were moderate, obtaining an overall F1 score of 0.51 points, with better recall than precision with 0.69 and 0.57 respectively. By evaluating the proposal by groups, a difference in precision was observed between groups 1 (older people), 2 (people with intellectual disabilities) and 3 (control users) with 0.57, 0.59 and 0.55 points, respectively. In turn, regarding the recall, there was a minor difference between groups, with 0.68 points for Group 1, 0.69 points for Group 2 and 0.69 points for Group 3.

**Table 6:** Result metrics for both groups in Task 1 where ID = User Id, AC = Acuraccy, PR = Precision and Group 1: older people, Group 2: people with intellectual disabilities and Group 3: control users.

**GROUP 1**					**GROUP 2**					**GROUP 3**				
**ID**	**AC**	**PR**	**RC**	**F-1**	**ID**	**AC**	**PR**	**RC**	**F-1**	**ID**	**AC**	**PR**	**RC**	**F-1**
1	0.57	0.78	0.52	0.40	16	0.56	0.52	0.65	0.40	31	0.56	0.52	0.59	0.42
2	0.58	0.78	0.53	0.42	17	0.63	0.59	0.77	0.54	32	0.60	0.55	0.73	0.47
3	0.68	0.74	0.65	0.63	18	0.60	0.55	0.73	0.47	33	0.59	0.55	0.73	0.46
4	0.77	0.80	0.75	0.75	19	0.59	0.55	0.69	0.47	34	0.59	0.54	0.71	0.44
5	0.70	0.74	0.68	0.67	20	0.61	0.57	0.70	0.51	35	0.59	0.55	0.73	0.46
6	0.61	0.70	0.57	0.51	21	0.59	0.56	0.58	0.53	36	0.56	0.51	0.78	0.37
7	0.58	0.78	0.53	0.42	22	0.65	0.61	0.70	0.58	37	0.71	0.70	0.72	0.70
8	0.56	0.78	0.51	0.37	23	0.59	0.56	0.60	0.53	38	0.58	0.53	0.78	0.42
9	0.59	0.68	0.54	0.45	24	0.65	0.61	0.77	0.56	39	0.57	0.52	0.68	0.42
10	0.56	0.58	0.51	0.40	25	0.65	0.61	0.75	0.57	40	0.55	0.50	0.53	0.37
11	0.63	0.80	0.58	0.52	26	0.66	0.62	0.78	0.58	41	0.63	0.59	0.70	0.54
12	0.59	0.65	0.54	0.46	27	0.64	0.61	0.69	0.58	42	0.59	0.54	0.79	0.43
13	0.53	0.51	0.51	0.48	28	0.67	0.64	0.75	0.62	43	0.56	0.52	0.65	0.40
14	0.56	0.78	0.51	0.37	29	0.65	0.61	0.77	0.56	44	0.63	0.59	0.77	0.54
15	0.63	0.73	0.59	0.53	30	0.62	0.59	0.65	0.56	45	0.59	0.54	0.71	0.44
**GROUP 1 SCORES**					**GROUP 2 SCORES**					**GROUP 3 SCORES**				
**ID**	**AC**	**PR**	**RC**	**F-1**	**ID**	**AC**	**PR**	**RC**	**F-1**	**ID**	**AC**	**PR**	**RC**	**F-1**
ALL	0.61	0.57	0.68	0.51	ALL	0.62	0.59	0.69	0.54	ALL	0.59	0.55	0.69	0.47
**OVERALL SCORE**														
**ACURACCY**				**PRECISION**				**RECALL**				**F1**		
0.61				0.57				0.69				0.51		

**Fig 3:**
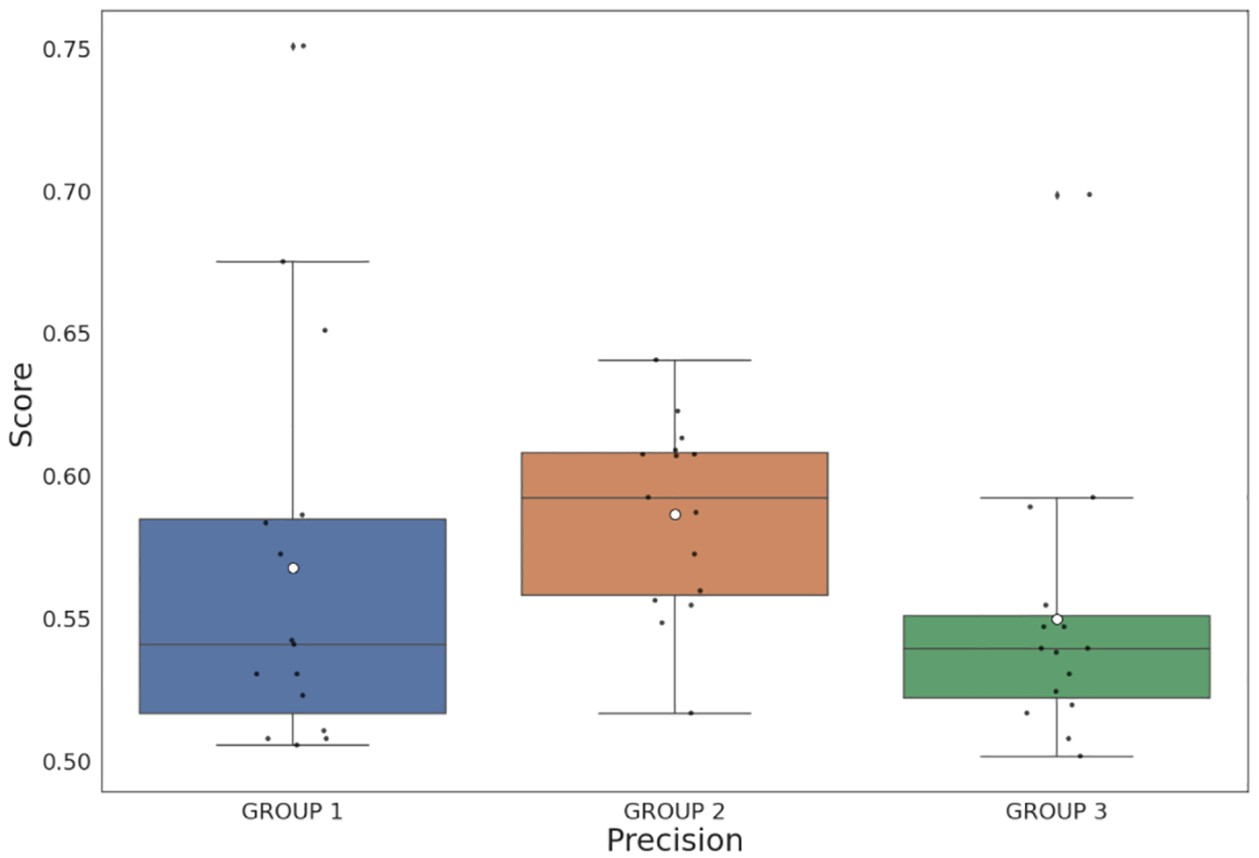
Precision scores among every participant divided into groups, where Group 1: older people, Group 2: people with intellectual disabilities and Group 3: control users.

Fig 3 shows a comparison of the precision scores between the study groups, where Group 2 (people with intellectual disabilities) achieved better results than Group 1 (older people) and Group 3 (control users). This indicates that the proposed CWI model achieved a higher number of quality predictions for people with intellectual disabilities than for older people and control users by getting a higher number of true positives. Although the difference in scores between the groups is minimal (about 0.02 points with Group 1 and 0.04 points with Group 3), this suggests that the proposal makes higher quality predictions for people with intellectual disabilities. Statistically comparing the precision between groups, the corpus was shown to be more beneficial for people with intellectual disabilities (Group 2) compared to older people in Group 1 (Wilcoxon test, *P* = 0.002) and control users in Group 3 (Wilcoxon test, *P* = 0.03).

**Fig 4:**
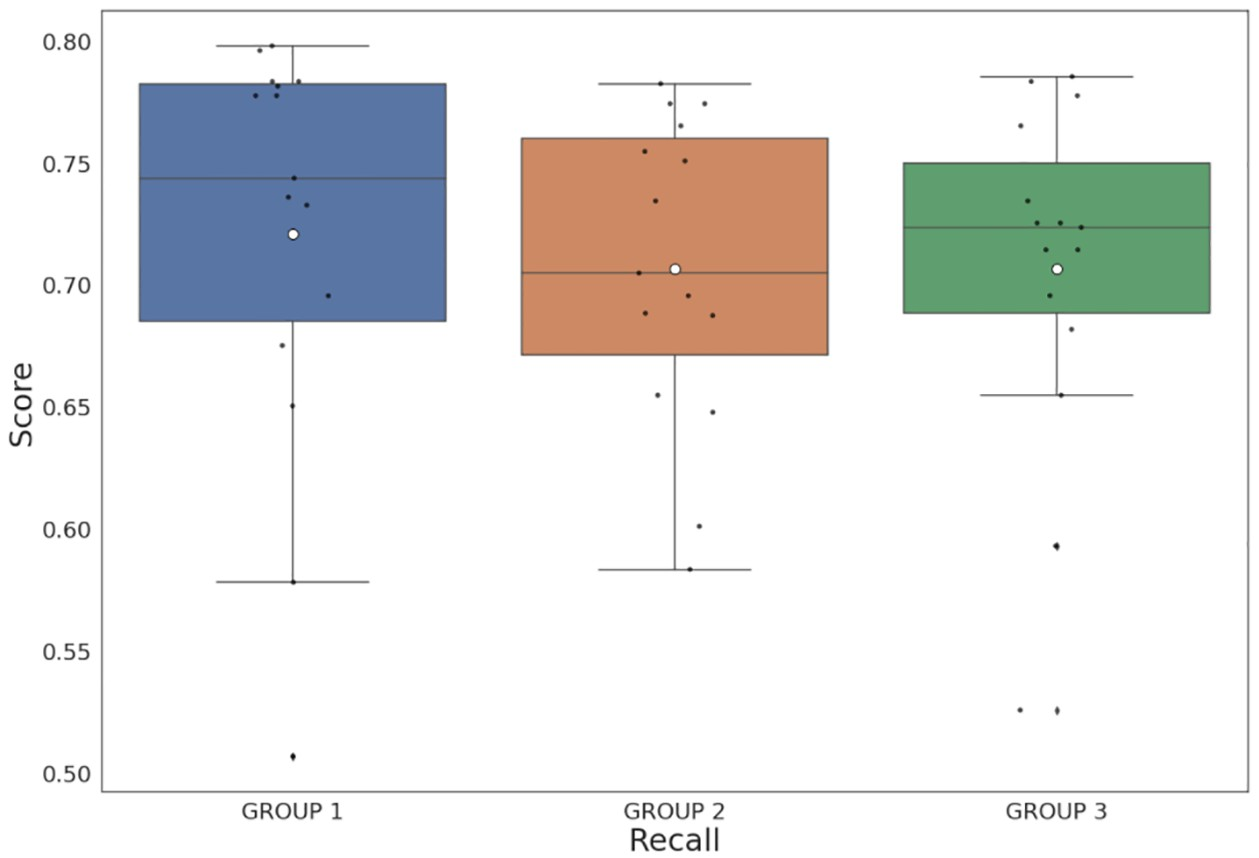
Recall scores among every participant divided by groups, where Group 1: older people, Group 2: people with intellectual disabilities and Group 3: control users.

On the other hand, when analyzing recall scores, an increase was noted in comparison to precision. Fig 4 compares the recall scores of the study groups, where a greater dispersion of the data is clearly seen in the Group 1 and Group 2 than in the Group 3. This metric is important for this study as the corpus seeks to cover as many terms as possible when providing cognitive language support to people with intellectual disabilities and the elderly. In contrast to precision, the corpus provides greater coverage for older people (Group 1) compared to control users in Group 3 (Wilcoxon test, *P* = 0.02).

**Fig 5:**
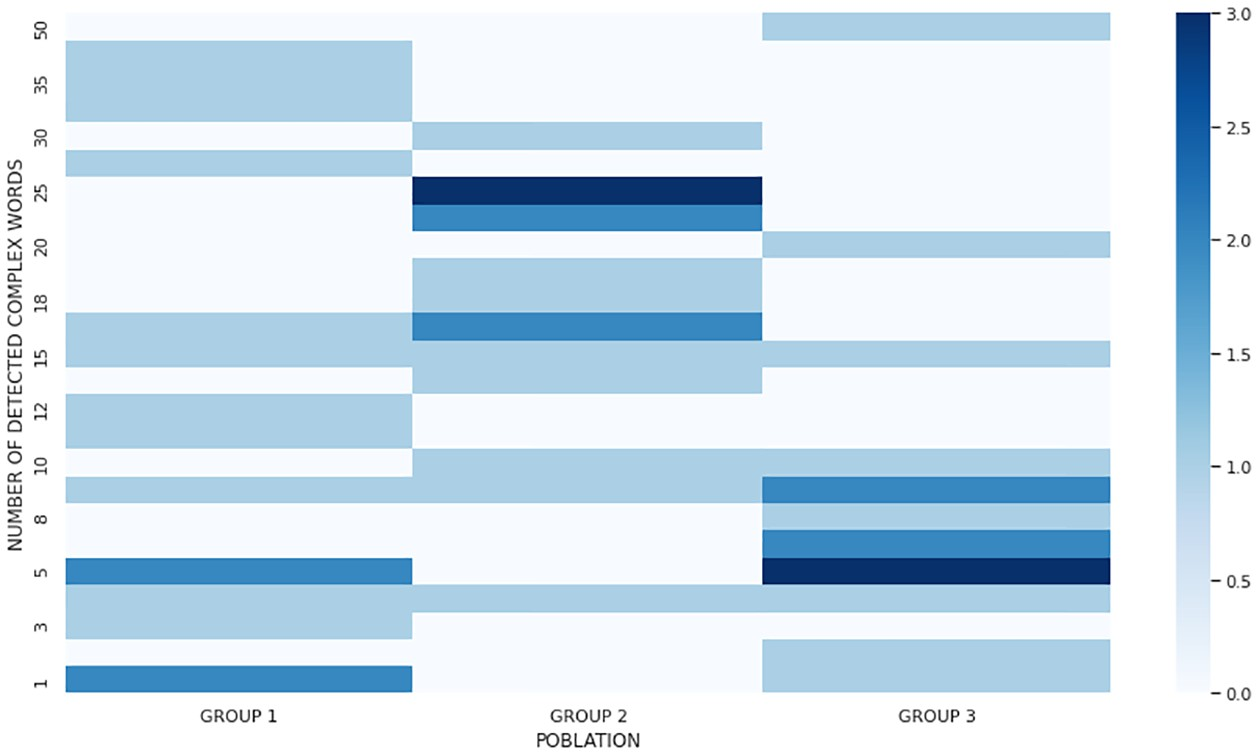
Number of detected complex words, divided by groups, where Group 1: older people, Group 2: people with intellectual disabilities and Group 3: control users.

In addition, Fig 5 presents the number of words that each participant considered complex, divided by groups. Most users in groups 1 and 3 are concentrated in the lower part of the graph where they detected a lower number of complex words (between 1 to 10 words across all sentences) and with additional values scattered across the graph. On the other hand, users with intellectual disabilities (Group 2), concentrated in a higher part of the graph by detecting a higher number of complex words, consequently supporting the precision and recall metrics described above.

Results achieved in CWI task do not seem very promising. We believe this could be related to ambiguity being greater in the case of open domain texts than in a restricted domain. Most research in NLP is devoted to solve the problem of ambiguity; NLP systems built to understand natural language only perform adequately in the domain for which they are designed and trained [67, 68], because the terminology is narrowed to a topic. Moreover, Gale et al. [69] showed that the sense of a target word is highly consistent within a given document (one meaning per discourse) and this reduces the number of synonyms of words in texts; this is comparable to the reduction of synonyms if texts of a restricted domain are considered. Nevertheless, simplification mechanisms are needed for information websites, such as news sites, that people access in search of information from a wide range of domains, hence the motivation for developing the Easier corpus. Moreover, experimentation with users is extraordinarily complex as it is carried out with subjective questions that measure how complex a word is for each person.

Related to the second task, the quality of the synonym dataset was evaluated and, as described above, each participant was asked to evaluate three candidate substitutes for each of the 15 sentences of the study. Table 7 shows three types of results divided by groups, the first where the number of users who accepted at least one of the candidates presented for each sentence is recorded, the second which records the number of users who accepted at least two of the candidates presented for each sentence and the last one being the most rigorous one that counts the number of cases where all candidates were accepted by instance.

**Table 7:** Task2: Number of cases where at least one candidate, two candidates and all candidates were ranked as correct, sorted by groups and sentences where Grp 1: older people, Grp 2: people with intellectual disabilities and Grp 3: control users.

	At least one candidate ranked as correct			At least two candidates ranked as correct			All candidates ranked as correct		
Sentence-ID	Grp 1 (N:15)	Grp 2 (N:15)	Grp 3 (N:15)	Grp 1 (N:15)	Grp 2 (N:15)	Grp 3 (N:15)	Grp 1 (N:15)	Grp 2 (N:15)	Grp 3 (N:15)
S1	15	15	15	11	12	13	7	9	5
S2	15	15	14	8	10	7	7	7	3
S3	15	15	14	10	9	9	8	8	4
S4	15	15	15	12	10	12	8	7	8
S5	14	15	14	12	11	10	8	10	5
S6	15	15	15	11	11	14	11	9	9
S7	14	15	14	10	11	10	8	7	2
S8	14	15	13	10	11	5	7	8	2
S9	15	14	14	11	10	11	8	7	5
S10	15	15	15	13	12	11	10	10	5
S11	15	14	14	12	11	13	10	8	6
S12	15	15	15	12	11	13	10	9	7
S13	14	15	14	10	10	7	9	8	4
S14	15	15	13	9	11	8	9	5	4
S15	15	14	14	11	6	9	9	5	3
**MEAN**	14.73	14.80	14.20	10.80	10.40	10.13	8.6	7.8	4.8
**ACCEPTANCE (%)**	98	99	95	72	69	68	57	52	32

Regarding the first result, an almost perfect percentage of acceptance was achieved for groups 1 (older people) and 2 (people with intellectual disabilities), with an acceptance percentage of 98% and 99% respectively. On the other hand, control users had a lower but close acceptance rate of 95%, mainly because this group of users does not represent the target user of the corpus. Therefore, this implies that the corpus greatly helps to reduce the level of complexity of the sentences, at least with a suggested candidate, and although a good acceptance was achieved in both groups, the group with intellectual disability was the one that received the most benefit. Later, more rigorous tests were carried out, where at least two candidates had to be accepted, obtaining in this case a higher percentage of acceptance of Group 1 than Group 2 with 72% and 69% respectively. Similarly, the acceptance rate of Group 3 dropped to 68%. Finally, when evaluating user responses in scenarios where all candidates were to be accepted, acceptance percentages of 57%, 52% and 32% were obtained for groups 1, 2 and 3, respectively.

Concerning the second task, statistical significance tests were performed to understand these results, where it was confirmed that the synonyms provided by the corpus help the population made up of older people in Group 1 and people with intellectual disabilities in Group 2 (Fisher test, *P* = 0.03), complementing the results shown in Table 7.

Later, these results were analyzed in relation to the education and reading level of each population. For example, the results showed statistically that the help of synonyms depended on the reading level of older users (Chi-square, *P* = 0.01).

**Fig 6:**
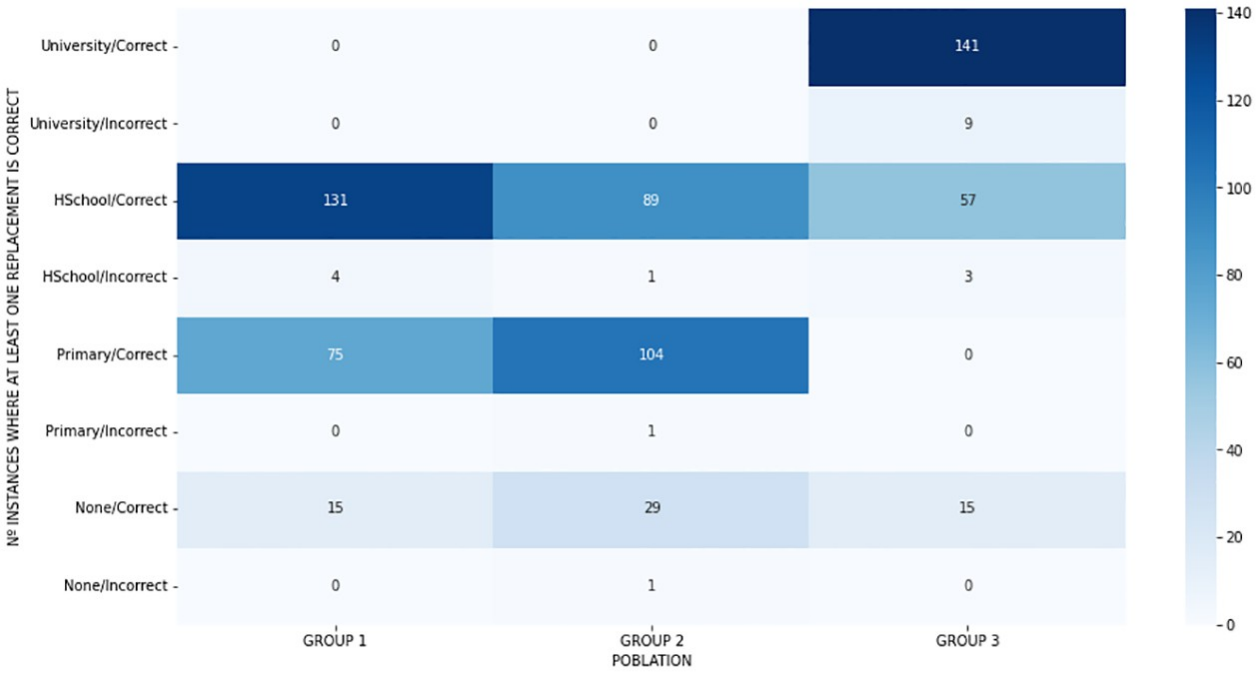
Number of instances where at least one substitute was taken as correct of incorrect, divided by group and education level, where Group 1: older people, Group 2: people with intellectual disabilities and Group 3: control users.

A similar example is shown in Fig 6 which divides the cases in which at least one substitution was accepted and the cases in which none was accepted, divided by group and educational level. For Group 1 (older people) there is a high number of substitutions accepted in participants with a high school level of education and a high number of acceptance for primary level of education for Group 2 (people with intellectual disabilities). It is worth mentioning that there is a higher concentration of participants with these levels of education for each group. For this same reason, there are cases in which the number of acceptances is low, as in the university level, which only had participants in Group 1.

## Conclusions

This article introduces the EASIER corpus, which compiles a total of 260 Spanish documents of different topics annotated with complex words and synonyms. The EASIER corpus provides support for NLP methods to face lexical simplification in Complex Word Identification (CWI) and Substitute Generation/Selection (SG/SS) tasks. As a result, approximately 8,100 complex words were gathered. Additionally, it contains approximately 5,100 words for which at least one synonym was proposed. This corpus was built thanks to the annotation and evaluation of linguistic experts, who are specialised in easy-to-read and plain language guidelines. Sixteen annotation guidelines to discern between complex and simple words are also defined.

The CWI dataset evaluation showed moderate IAA with a Fleiss Kappa coefficient of 0.641. On the other hand, an evaluation of this dataset with both target and control users, achieved a moderate overall F1-score of 0.51 points. However, since this corpus seeks to meet the needs of people with cognitive disabilities, greater importance was given to the recall metric, which was 0.68 and 0.69 points for older people and people with intellectual disabilities, respectively. Finally, a range of significance tests were also performed to confirm the corpus support between populations.

Concerning the moderate IAA in complex word annotation tasks, it is important to highlight that tasks that require more interpretation of texts do not obtain a high agreement among annotators [63]. A high IAA is an indicator that the task is well defined and other annotators could replicate the work. Specifying if a word or phrase is a complex term is a subjective task, which influences the IAA value. In addition, the fact that an annotator has a high IAA certainly does not mean that the annotations are correct. It means that annotators have equally interpreted the guidelines. Bayerl and Paul [70] analyzed several factors that could influence IAA through different labeled corpora providing some recommendations to improve IAA like using few categories, recruiting annotators with the same level of domain expertise and providing training to them. To gain confidence in the integrity of annotations, they suggest having larger groups of annotators considering the criticality of tasks. In annotation tasks as the one described in this study, having expert and trained annotators in plain language and easy-to-read guidelines is essential.

The evaluation of the SG/SS dataset showed positive results. Out of the 1,026 synonyms analysed, 987 were scored as well-defined by one annotator and 913 by the other one. The same people from the previous study evaluated a portion of the synonym dataset. Near-perfect results were obtained for cases where at least one synonym was accepted (out of 3), and moderate-to-good results were obtained for scenarios where two or more synonyms were accepted. As in the former dataset study, statistical tests were performed in order to confirm various hypotheses.

This corpus is publicly available and currently being used in the EASIER platform. It has been created as a resource to assist both researchers and companies in carrying out simplification processes, with the added value that has been validated by people with disabilities.

The EASIER corpus provides support for lexical simplification processes in a generic domain; lexical simplification of domain-independent texts is an extremely complex task, hence some of its moderate results. An extension of this resource will be developed for restricted domains (e.g., eGovernment, legal and health texts, among others) in future work. In addition, over the years, different scales have been proposed to evaluate complexity in texts [66], so the incorporation of new complexity scales (non-binary scale) will be evaluated.

## Supporting information

S1 Table Annotation criteria examples. (PDF)

S2 Table CWI dataset instance examples. (PDF)

S3 Table SG/SS dataset instance examples. (PDF)
