# Supplementary material for: EASIER corpus: A lexical simplification resource for people with cognitive impairments
Source: PLoS One. 2023 Apr 12;18(4):e0283622. doi: 10.1371/journal.pone.0283622 (PMC10096182; doi:10.1371/journal.pone.0283622)
Supplement: S1 Table — (PDF) [file pone.0283622.s001.pdf]

S1 Table. Annotation Criteria examples.

Sentence	Word	Criteria
El Ministerio de Sanidad, Consumo y Bienestar Social ha remitido hoy los informes provisionales de cinco de las 66 técnicas sometidas a evaluación dentro del Plan de Protección de la Salud frente a las Pseudoterapias. (Today, the Ministry of Health, Consumer Affairs and Social Well-being has issued the provisional reports on five of the 66 techniques evaluated by the Health Protection Plan against Pseudotherapies.)	Pseudoterapias (Pseudotherapies)	2, 3, 4
El punto de partida de este proceso fue un análisis exploratorio inicial de 138 técnicas o procedimientos, basado en una revisión de las publicaciones científicas (revisiones sistemáticas y ensayos clínicos). (The starting point of this process was an initial exploratory analysis of 138 techniques or procedures, based on a review of scientific publications (systematic reviews and clinical trials))	ensayos clínicos (clinical trials)	1, 2, 11, 12
Las instituciones que lo forman son la OMC, el Consejo General de Enfermería, el Consejo General de Colegios Oficiales de Farmacéuticos, AEMPS, SECA, el Consejo General del Trabajo Social, SEMG, semFYC, AEP, SEMERGEN, EUPATI, CERMI, la SEMI. (The institutions comprising this group are the WTO, the General Council of Nursing, the General Council of Pharmacists Associations, AEMPS, SECA, the General Council of Social Work, SEMG, semFYC, AEP, SEMERGEN, EUPATI, CERMI, and the SEMI.)	semFYC	5
... al tiempo que ha manifestado la necesidad de que el desarrollo del marco estratégico de su abordaje tenga en cuenta “la efectividad, la eficiencia, la accesibilidad, la seguridad y la atención centrada en el paciente”. (...while at the same time stating the need for the development of the approach of the strategic framework to take into consideration “the effectiveness, efficiency, accessibility, security and patient-focused care.”)	marco estratégico (strategic framework)	1, 2, 11, 12
El tercer eje se refiere al derecho, la ética y la dignidad de la persona y pretende mejorar los servicios, apoyos y prestaciones para avanzar en la atención a las personas en distintos ámbitos. (The third pillar refers to the right, ethics and dignity of the person and aims to improve services, support and benefits in order to improve care for people in different areas.)	Ética (ethics)	10
... así como recambios de componentes externos de implantes quirúrgicos (componentes externos del estimulador diafragmático o electroestimulador del nervio frénico). (... as well as the replacement of external surgical implant components (external components of the diaphragmatic stimulator or phrenic nerve electro-stimulator).)	Diafragmático (diaphragmatic)	2, 3, 4
“España está trabajando en este objetivo en el marco de la iniciativa Connecting Europe Facility”, ha señalado la ministra. (“Spain has been working on this goal within the framework of the Connecting Europe Facility initiative”, the minister pointed out.)	Connecting	6
Desde los recogidos por encuestas de salud de la población, que obtienen periódicamente información en cerca de 30.000 hogares; las altas hospitalarias o el seguimiento en la Atención Primaria de una cohorte de 4,7 millones de personas. (From data collected by population health surveys, which periodically obtain information from around 30,000 households; hospital discharges or the follow-up of a cohort of 4.7 million people in primary care.)	Periódicamente (periodically)	2, 14
Una mirada a la indumentaria tradicional. (A look at traditional clothing.)	Indumentaria (clothing)	15
El spot de la campaña se emite en televisiones autonómicas y estatales desde el 14 al 24 de noviembre. (The campaign spot will be broadcast on both state and regional television stations from November 14th to the 24th.)	spot	1, 6
Costar un ojo de la cara. (Cost an arm and a leg.)	“Entire expression”	8
Debes tener un tornillo suelto. (You must have a screw loose.)	tener (have)	9
