# Supplementary material for: EASIER corpus: A lexical simplification resource for people with cognitive impairments
Source: PLoS One. 2023 Apr 12;18(4):e0283622. doi: 10.1371/journal.pone.0283622 (PMC10096182; doi:10.1371/journal.pone.0283622)
Supplement: S2 Table — (PDF) [file pone.0283622.s002.pdf]

S2 Table. CWI dataset instance examples

Doc ID	Sentence ID	Sentence	Start offset	End offset	Word	Label
1	136	La importancia de leer bien el etiquetado antes de comprar un alimento. (The importance of carefully reading the labelling before purchasing foodstuffs.)	3	14	Importancia (importance)	0
1	136	La importancia de leer bien el etiquetado antes de comprar un alimento. (The importance of carefully reading the labelling before purchasing foodstuffs.)	18	22	Leer (reading)	0
1	136	La importancia de leer bien el etiquetado antes de comprar un alimento. (The importance of carefully reading the labelling before purchasing foodstuffs.)	31	41	Etiquetado (labelling)	1
1	136	La importancia de leer bien el etiquetado antes de comprar un alimento. (The importance of carefully reading the labelling before purchasing foodstuffs.)	51	58	Comprar (purchasing)	0
1	136	La importancia de leer bien el etiquetado antes de comprar un alimento. (The importance of carefully reading the labelling before purchasing foodstuffs.)	62	70	Alimento (foodstuffs)	0
