# Supplementary material for: EASIER corpus: A lexical simplification resource for people with cognitive impairments
Source: PLoS One. 2023 Apr 12;18(4):e0283622. doi: 10.1371/journal.pone.0283622 (PMC10096182; doi:10.1371/journal.pone.0283622)
Supplement: S3 Table — (PDF) [file pone.0283622.s003.pdf]

S3 Table. SG/SS dataset instance examples

Doc ID	Word ID	Word	Sentence	Proposed Substitutions
1	167	Etiquetado (labelling)	La importancia de leer bien el etiquetado antes de comprar un alimento. (The importance of carefully reading the labelling before purchasing foodstuffs.)	Letrero (sign), inscripción (inscription), rótulo (banner)
1	168	Etiqueta (label)	La campaña 'Esta Navidad… Que tu mesa se vista de etiqueta' con el objetivo de recordar a la población la información que debe tener en cuenta a la hora de comprar alimentos envasados o a granel así como los datos que deben figurar en las páginas web que venden online (The "This Christmas… dress up your table*" campaign aims to remind people of what information they need to keep in mind when purchasing packaged foodstuffs or in bulk, as well as the information that must be included on webpages that sell online. *in the original Spanish, the name of this campaign uses a play on words with the different meanings of "etiqueta", which can mean either formal dress or a label.)	Ceremonia (ceremony), protocolo (protocol)
1	169	Envasados (packaged)	La campaña 'Esta Navidad… Que tu mesa se vista de etiqueta' con el objetivo de recordar a la población la información que debe tener en cuenta a la hora de comprar alimentos envasados o a granel así como los datos que deben figurar en las páginas web que venden online (The "This Christmas… dress up your table*" campaign aims to remind people of what information they need to keep in mind when purchasing packaged foodstuffs or in bulk, as well as the information that must be included on webpages that sell online. *in the original Spanish, the name of this campaign uses a play on words with the different meanings of "etiqueta", which can mean either formal dress or a label.)	Empaquetados (packaging)
1	170	a granel (in bulk)	La campaña 'Esta Navidad… Que tu mesa se vista de etiqueta' con el objetivo de recordar a la población la información que debe tener en cuenta a la hora de comprar alimentos envasados o a granel así como los datos que deben figurar en las páginas web que venden online (The "This Christmas… dress up your table*" campaign aims to remind people of what information they need to keep in mind when purchasing packaged foodstuffs or in bulk, as well as the information that must be included on webpages that sell online. *in the original Spanish, the name of this campaign uses a play on words with the different meanings of "etiqueta", which can mean either formal dress or a label.)	Suelto (loose), sin envase (without packaging)
1	171	online	La campaña 'Esta Navidad… Que tu mesa se vista de etiqueta' con el objetivo de recordar a la población la información que debe tener en cuenta a la hora de comprar alimentos envasados o a granel así como los datos que deben figurar en las páginas web que venden online (The "This Christmas… dress up your table*" campaign aims to remind people of what information they need to keep in mind when purchasing packaged foodstuffs or in bulk, as well as the information that must be included on webpages that sell online. *in the original Spanish, the name of this campaign uses a play on words with the different meanings of "etiqueta", which can mean either formal dress or a label.)	en línea (online), conectado a Internet (connected to the Internet)
